# Integration of resting-state and stimulus-fMRI uncovers reduced network flexibility in post-surgical pain

**DOI:** 10.1038/s41598-026-51946-5

**Published:** 2026-05-19

**Authors:** Bruno Pradier, Esther Pogatzki-Zahn, Cornelius Faber, Daniel Segelcke

**Affiliations:** 1 Department of Anesthesiology, Intensive Care and Pain Medicine, University Hospital Muenster, Albert-Schweitzer-Campus 1, A1, 48149 Muenster, Germany; 2 Department of Clinical Radiology, Translational Research Imaging Center, Munster, Germany

**Keywords:** Biological techniques, Neuroscience

## Abstract

Functional MRI (fMRI) provides complementary insights into brain network organization during rest (rs-fMRI) and external stimulation (t-fMRI). While rs-fMRI reveals intrinsic connectivity patterns, t-fMRI reflects stimulus-dependent network responses. However, how these states relate to each other in animal disease models remains incompletely understood. We performed sequential acquisition of rs-fMRI and t-fMRI during low- and high-intensity mechanical paw stimulation in a rat model of post-surgical pain (PSP) and SHAM controls. Functional connectivity and network organization were analyzed using network-based statistics and graph-theoretical metrics. In addition, supervised classification of node-level network parameters was performed using linear discriminant analysis (LDA), and regional contributions to network separation were quantified using Mahalanobis distance metrics. Global connectivity strength and small-world organization were preserved across different imaging conditions in both groups, indicating stable network topology. However, multivariate classification revealed clear modality-dependent network signatures in SHAM animals that were markedly reduced in PSP. Region-wise Mahalanobis analyses showed that stimulus-related network shifts were more heterogeneous in SHAM animals, with higher dispersion and recurrent regional “hotspots”, whereas PSP animals exhibited more uniform and spatially diffuse responses. These findings indicate that post-surgical pain primarily affects the flexibility of regional network reconfiguration rather than global topology. Combining rs- and stimulus-based fMRI thus provides complementary insight into disease-related network alterations that are not detectable from resting-state data alone.

**Supplementary Information:**

The online version contains supplementary material available at 10.1038/s41598-026-51946-5.

## Introduction

Functional Magnetic Resonance Imaging (fMRI) is a widely used tool in neuroscience, enabling the investigation of brain states regarding activity and connectivity. Resting-state fMRI (rs-fMRI) has become popular due to its ease and efficiency of acquisition, allowing the exploration of the intrinsic functional connectivity of the brain without the need for a specific extrinsic task in both humans and animal models^1–4^. These intrinsic network patterns provide a baseline architecture upon which moment-to-moment responses to external stimuli are dynamically shaped^5,6^, and studies have shown that resting-state connectivity can partially predict inter-individual variation in task-evoked activity^7,8^.

Despite these links, task-based fMRI (t-fMRI) provides a complementary way to probe how the brain responds to specific cognitive and sensory stimuli, directly mapping the neural systems recruited during controlled experimental conditions. Supraspinal task-evoked networks are often temporally coherent and congruent with those observed at rest, yet may show context-dependent shifts in specific subnetworks^9–11^. Importantly, in many contexts functional connectivity (FC) measured during tasks can better capture variance related to supraspinal activations and behavioral phenotypes than connectivity derived from rest^12,13^. This added predictive value underscores the importance of considering both states together rather than in isolation, particularly when probing disease-specific changes in network dynamics. Notably, such effects are not necessarily reflected in global graph measures, which may remain largely conserved while region-specific or multivariate signatures change.

In animal models, the application of t-fMRI diverges from human studies due to the inability to perform cognitive tasks, especially under anesthesia, which is known to affect neurovascular coupling. Instead, sensory stimulation (e.g. somatosensory, visual, or olfactory) is frequently used to probe different brain circuits^14–17^, including the use of graded stimulus intensities to sample response dynamics. This approach aligns with the fundamental principles of t-fMRI but is adapted to the practical and physiological constraints of animal models. Both rs- and t-fMRI are now widely used to delineate functional networks in rodents^18^. However, systematic comparison of these two states remains limited, particularly within-subject under matched conditions, under matched acquisition conditions, and identical anesthetic regimens. Such comparisons are particularly relevant in pathological conditions, where disease-related shifts in network operating points may constrain state differentiation and reduce response flexibility. Thus, contrasting rs- and t-fMRI is informative to assess baseline setpoint, dynamic range across stimulus intensities, and potential constraints on network adaptations.

In this secondary analysis, we test whether a disease state (post-surgical pain; PSP) alters the relationship between rs- and sensory stimulation fMRI, using low- and high-intensity mechanical stimulation as a probe of supraspinal hypersensitivity. Using complementary analytical approaches, we find that global network topology was broadly similar across conditions, whereas regional multivariate analyses revealed modality- and model-dependent patterns. Overall, the results are consistent with a constrained differentiation of network responses across stimulus intensities under PSP, in line with the concept of a disease-induced state shift.

## Materials and methods

### Study information

This report presents a secondary analysis of previously published data^16^. The original study examined resting-state brain networks in a rat model of post-surgical pain (PSP) compared with sham controls (SHAM) and their modulation during mechanical stimulation. Here, we focused on differences between intrinsic (rs-fMRI) and stimulus-evoked (t-fMRI) network organization under low- and high-intensity stimulation. Data acquisition and preprocessing followed the original study, while the present analysis extends the workflow by applying multivariate classification of node-level graph-theoretical parameters using linear discriminant analysis (LDA) and Mahalanobis-based separation metrics.

### Animals

All animal experiments were performed according to the German *Tierschutzgesetz* and were approved by the State Agency for Nature, Environment and Consumer Protection North Rhine-Westphalia (LANUV, approval 84-02.04.04.2014.A347). In total, 25 male Sprague–Dawley rats (180–250 g, Charles River Laboratories, Sulzfeld, Germany) were housed in pairs (sham–sham or incision–incision) in individually ventilated Double Decker cages (SEALSAFE PLUS Rat GR1800; Tecniplast). Animals were kept on a 12/12 h light/dark cycle with *ad libitum* access to standard chow and water, and cages included enrichment (wood for gnawing, a paper shelter/tube, and paper nesting material). At the study end, animals were euthanized by gradual-fill CO₂ inhalation (displacement rate 20–30% chamber volume/min) until 1 min after respiratory arrest, followed by cervical dislocation as a secondary method. Reporting was conducted in accordance with ARRIVE 2.0 guidelines^19^.

### Incisional pain model

Male rats underwent a procedure for plantar incision as previously described^20^. Briefly, under isoflurane anesthesia (1.5–2.5 S%, in 100% oxygen delivered via a nose cone, *n* = 18), a 1 cm skin and fascia incision, starting 0.5 cm below the right heel, of the plantar aspect was performed. The muscle was exposed and incised in the middle and stretched. The wound was then closed with two skin sutures (Prolene 5 − 0). Sham-operated (same anesthesia duration, 15 min, without any surgical intervention, *n* = 7) animals served as controls. The rats were randomly selected to receive a plantar incision or a Sham surgery. No perioperative analgesics or antibiotics were administered.

### BOLD fMRI and mechanical stimulation post-surgical injury

One day after hind paw incision, we performed blood-oxygen-level-dependent (BOLD) fMRI experiments as recently published^21,22^. We used two intensities (low-intensity mechanical stimulation (LMS) = 45 g and high-intensity mechanical stimulation (HMS) = 90 g) for mechanical probing of the right hind paw with von Frey monofilaments. Stimulus-induced scans followed a block design comprising 10 s OFF–10 s ON–10 s OFF intervals repeated 20 times (total 600 s). During each ON period, the von Frey filament was applied at 1 Hz with an approximate contact duration of 0.5 s. fMRI measurements were conducted on a 9.4 T Bruker Biospec 94/20 small animal scanner (Bruker Biospin GmbH, Ettlingen, Germany) using a single loop surface coil (Bruker). Adjustments were conducted and anatomical images were acquired (RARE: TR/TE = 4000/12.5 ms; RARE factor = 8; FOV = 28 × 26 mm^2^; 256 × 256; 16 slices; slice thickness = 1.2 mm; 4 averages), followed by shimming using MAPSHIM (Bruker) over the entire brain. Anesthesia was started with isoflurane alone and subsequently switched to medetomidine sedation (bolus 0.04 mg/kg and infusion 0.05 mg/kg∙hr) as described recently^22,23^. Respiration rate and body temperature were constantly monitored and maintained within a physiological range. fMRI scans were acquired with a standard GE-EPI sequence (TR/TE:1000/18ms, 1.2 mm thick, FOV 28*26mm2, matrix 80*80, 600 images, 10 min) at rest (rs-fMRI) and during mechanical stimulation (t-fMRI). Baseline activity was measured 40 min after stopping isoflurane anesthesia.

### fMRI-data processing

Preprocessing of both, rs- and t-fMRI-data was performed with SPM (see below) and included slice time and motion correction (3D with trilinear/sinc interpolation), spatial (Gaussian, full width at half maximum (FWHW) 0.6 mm) and temporal (Gaussian, FWHW 3 s) smoothing. A 0.1 Hz low-pass filter was applied to the rs-fMRI data, followed by global signal mean removal via linear regression. Manual skull-stripping was performed on rs- and t-fMRI data from each animal. An atlas containing 136 brain regions based on the Franklin and Paxinos rat brain atlas^24^ was registered slice-wise (affine, 6 degrees of freedom) to individual EPI volumes of each rat. Regions of interest (ROI) represent specific brain structures from 10 functional groups: sensory cortex (SC), association cortex (AC), thalamus (Th), sensory input (SI), limbic system (LS), link to the limbic system (LLS), limbic output (LO), basal ganglia (BG), motor cortex and cerebellum (MO, Suppl. Table 1).

### Quality control

Quality control was carried out as described earlier^16^. Briefly, to ensure high-quality rs- and t-fMRI data, rigorous quality control measures were implemented. Initial visual inspection and rating of EPI image quality on a scale of 1 to 10 were conducted blindly. Scans with sudden frame displacements exceeding 50% of voxel size were excluded (5 datasets). Multi-seed-correlation analysis with graph theory was performed, and correlation matrices of FC were visually inspected to flag aberrant patterns. Objective quantification of FC quality was achieved by extracting parameters such as interhemispheric connectivity and FC strength (converted to Z-values). An upper threshold of 6000 for Z-values was determined based on histogram distribution to exclude scans showing aberrant FC patterns (9 scans). Finally, single FCs known for high specificity in brain networks were extracted to serve as physiological controls, with expected low correlation between medial and mediolateral networks and high correlation for interhemispheric connectivity in the S1 cortex. Following this quality control group sizes were as follows: RS/LMS-SHAM: *n* = 6, HMS-SHAM: *n* = 7, RS-PSP: *n* = 14, LMS/HMS-PSP: *n* = 17.

### Functional connectivity analysis

Utilizing the multi-seed region approach (MSRA), FC was assessed in resting-state and stimulus-induced fMRI data, as detailed in earlier studies^23,25^. Briefly, a seed was defined for each brain structure, which comprised five voxels in its center of mass. Next, the average time course of each seed region was band-pass filtered (0.01–0.1 Hz) and correlated with the time course of every voxel in the brain, and the average correlation value of significantly correlating voxels (as determined by FDR with q = 0.05) per brain structure was used to create animal-specific correlation matrices. These correlation matrices were averaged for each experimental condition. For statistical analysis, correlation coefficients (Pearson’s R) values were first Fisher z-transformed and then analyzed with z-test statistics. The resulting p-values (*p* < 0.05) were corrected for multiple comparisons using FDR (q = 0.05) according to Benjamini and Yekutieli^26^. To statistically evaluate the effect of the stimulation modality, we used the network-based statistics (NBS) as described recently^16^: a paired t-test was applied for two groups for each connection, and the largest components of interconnected significantly modulated connections (*p* < 0.05) were retained. Next, permutation testing was performed by randomly switching the sign of each pair (1000 iterations) to correct for multiple comparisons. The estimated multiplicity-adjusted p-value (p_FWE_) for an observed original component was the proportion of permutation samples in which the component size was greater than or equal to the size of the observed component (component size defined as the number of edges).

### Local graph-theoretical network and linear discriminant analysis

Local graph-theory parameters that characterize node connectedness (degree and strength), integration or segregation within a network (node-specific clustering coefficient and path length), and network importance (betweenness centrality, hub score, and authority) were calculated with MagnAn as described recently for each animal^25^. Consequently, each node was characterized by a complex, high-dimensional set of graph-theory (GT)-parameters. For dimensionality reduction and hence the selection of relevant features, a LDA was used for rs- and t-fMRI data sets. This analysis aimed to categorize nodes based on their group membership. LDA identifies linear combinations of variables that best distinguish between two or more groups^25^. These combinations create a new, simplified coordinate system, with dimensions ordered by their ability to differentiate between groups. Consequently, we used the first two LD-dimensions to visualize how nodes are grouped. Additionally, the feature loadings on these LD-dimensions highlighted the variables most critical for distinguishing between groups, essentially defining the unique bio-signature for each group. Finally, we calculated the Mahalanobis distance for each node across all LD dimensions between each pair of groups. A larger Mahalanobis distance shows a greater degree of separation of that brain region between groups.

### Node-wise multivariate segregation and regional “spikiness” analysis

To quantify how individual brain structures contributed to stimulus-related network reconfiguration in the multivariate latent space, we analyzed region-wise Mahalanobis distances derived from the LDA embedding of graph-theoretical parameters. For each brain region and animal, Mahalanobis distances (DMah) were computed relative to the group centroids in LD space. To specifically characterize stimulus-related changes in multivariate network structure, we calculated within-animal delta values representing the absolute difference between resting-state and stimulus conditions (dLMS/dHMS). These ΔMah values quantify the magnitude of network-state displacement for each brain region relative to the resting-state configuration. Group-level differences in the distribution of ΔMah values between SHAM and PSP animals were evaluated using the Kolmogorov–Smirnov (KS) test. To assess differences in dispersion (variance heterogeneity), we applied Levene’s test and Brown–Forsythe statistics, complemented by robust dispersion metrics such as the median absolute deviation (MAD). This approach allows detection of differences not only in mean separation but also in the spread of regional contributions within the multivariate space.

To identify brain regions that consistently acted as dominant contributors to stimulus-related network shifts, we performed a region-level hotspot analysis. For each animal and contrast (dLMS, dHMS), brain regions belonging to the top 10% of ΔMah values were identified. These peaks represent the strongest regional contributors to network-state displacement in the multivariate embedding. We then tallied, across animals within each group, how frequently each region appeared in these peak sets. This cross-animal frequency analysis quantifies the spatial localization and consistency of network segregation, enabling comparison of whether stimulus-driven network changes are characterized by focal regional spikes or more distributed patterns across brain regions.

All analyses were performed on available regions per animal. Missing values were handled using pairwise deletion, ensuring that Mahalanobis statistics were computed from all valid regional observations.

### Software and statistical analysis

Preprocessing of fMRI data and GLM analysis were performed with SPM12 in the MATLAB environment (The MathWorks, Inc., Natick, Massachusetts, USA). For all other fMRI data analysis steps, we used MagnAn 2.5 (BioCom, Uttenreuth), an IDL application designed for complex image processing and analysis, emphasizing MR imaging and graph theory. One-way ANOVAs were calculated with GraphPad Prism (8.0.2, San Diego, CA, USA). Multifactorial analyses of graph-theoretical (GT) parameters were performed in SPSS (IBM, NY, USA). For each metric (Strength, Degree, Cluster, Path, Between, Hub), we fit general linear models with Group (SHAM vs. INC) and Imaging condition (RS, LP, HP; or RS vs. LP/HP contrasts) as fixed factors. Distributional analyses of Mahalanobis deltas (ΔMah), including Kolmogorov–Smirnov and variance heterogeneity tests (Levene/Brown–Forsythe), as well as dispersion metrics and hotspot tallies, were implemented in Python using the NumPy and SciPy libraries.

## Results

We recently detailed processing differences between SHAM and PSP networks using different imaging modalities, including rs- and t-fMRI^16,22,25^. Our study centered on the contrasts among different imaging conditions to assess the additional benefit of t-fMRI versus solely rs-fMRI in rodents. We compared rs-fMRI brain networks to those obtained following low- and high-intensity mechanical stimulation in both experimental groups, respectively.

### Imaging conditions do not differ at the level of FC or global network properties

**Fig. 1:**
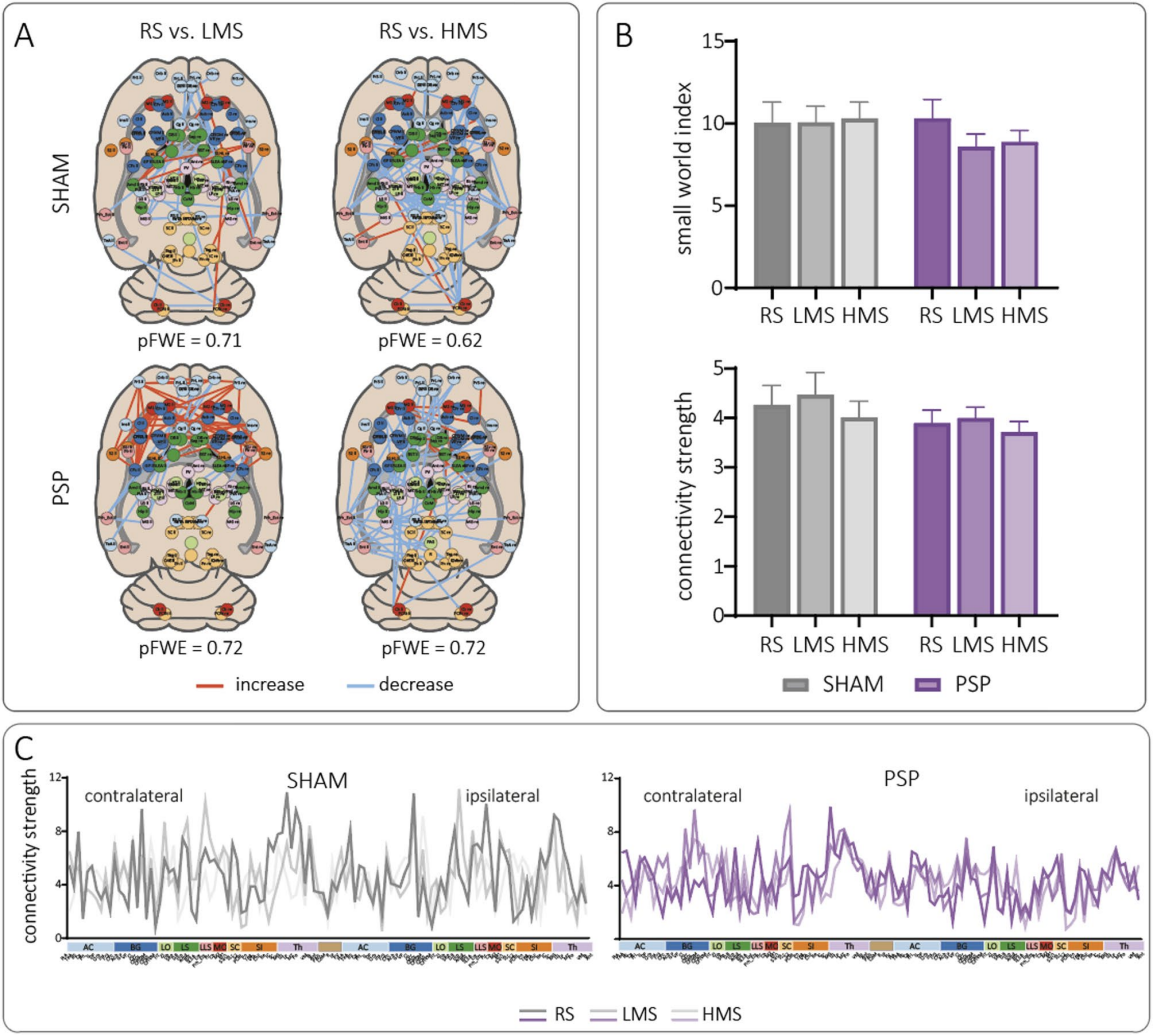
Comparison of functional imaging conditions at whole-brain and local network levels. **(A**) NBS analysis of functional connectivity matrices revealed no significant differences between resting-state (RS) and low- (LMS) or high-intensity (HMS) mechanical stimulation in SHAM (top) or post-surgical pain (PSP; bottom) animals. (**B**) Global network properties did not differ across modalities. The small-world index (top) and average FC strength (bottom) were unchanged between , LMS, and HMS conditions in both SHAM and PSP post-surgery (one-way ANOVA; n.s.). C) Node-level graph-theoretical metrics revealed substantial variability in local connectivity parameters (e.g., node strength; top). RS/LMS-SHAM: *n* = 6, HMS-SHAM: *n* = 7, RS-PSP: *n* = 14, LMS/HMS-PSP: *n* = 17. Node labels are readable in the separately downloadable high-resolution PDF of Fig. 1.

Using permutation testing within the Network-Based Statistic (NBS) framework, we compared functional connectivity (FC) between rs- and t-fMRI with low- or high-intensity mechanical stimulation. No significant FC differences were detected in SHAM (Fig. 1A, top) or in PSP animals (Fig. 1A, bottom). Although NBS is sensitive in identifying differences in individual FC, it does not account for the broader network architecture. Consequently, we analyzed the small-world index, reflecting the efficiency of information flow within a network (Fig. 1B, top). We observed no imaging condition-induced changes in this parameter in either SHAM or PSP animals (1-way ANOVA, SHAM: F_(2, 17)_ = 0.03 *p* = 0.98; PSP: F_(2, 46)_ = 0.64, *p* = 0.35). Similarly, we did not find modality-induced differences in FC strength (Fig. 1B, bottom, one-way ANOVA, SHAM: F_(2, 17)_ = 0.10 *p* = 0.71; PSP: F_(2, 46)_ = 0.11, *p* = 0.66).

Next, we applied a set of graph-theoretical metrics following our previously described workflow^16,25^ to explore processing at the network level of individual brain structures. These local parameters include node strength, degree, cluster coefficient, average path-length, betweenness centrality, and HITS-hub score. As reported previously, differences between SHAM and PSP became evident at this local level^16^. However, comparing imaging modalities (RS-LMS, RS-HMS), no differences were detected for local parameters in either SHAM or INC (all *p* ≥ 0.225, see Fig. 1C with connectivity strength as representative example), whereas only path length showed small, but statistically detectable condition effects (SHAM RS–LP: F_(1,1378)_ = 6.227, *p*=0.013; INC RS–HP: F_(1,3563)_ = 21.006, *p*<0.001; INC RS–LP: F_(1,3563)_ = 25.568, *p*<0.001).

These results demonstrate that the global brain network architecture remains largely unaffected by the imaging condition, with respect to individual FC, FC strength, or network efficiency.

### Multivariate classification reveals group and stimulus separation in network space

**Fig. 2:**
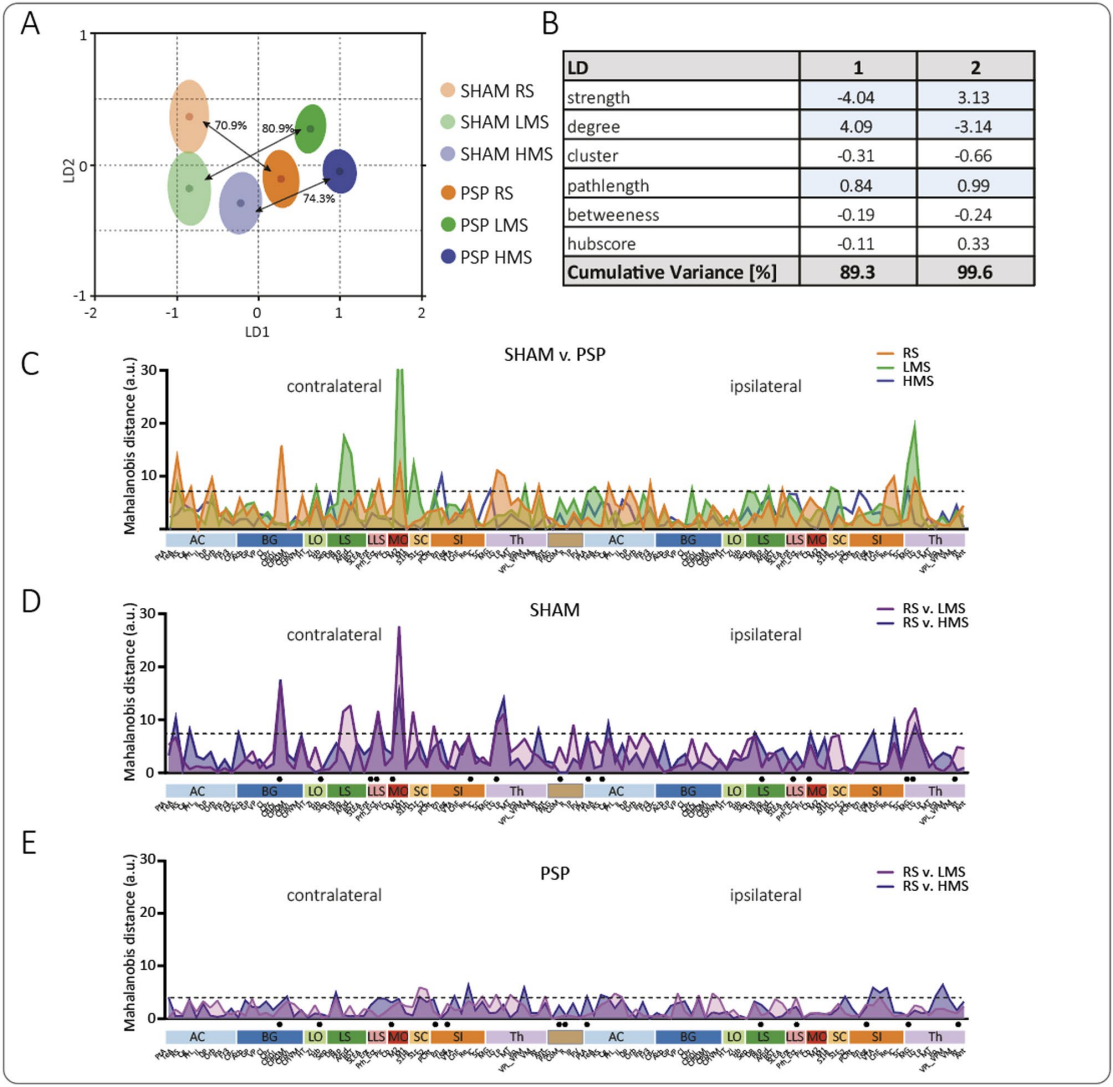
Supervised classification of network topology reveals modality- and model-specific signatures. (**A**) LDA based on node-level graph-theoretical metrics separated SHAM and PSP groups primarily along the first linear discriminant (LD1) and imaging conditions (rs- and t-MRI) along the second (LD2). Cross-validated classification achieved 70.9–80.9% accuracy for distinguishing imaging conditions across the combined dataset (SHAM vs. PSP), and 60.9–67.4% accuracy for distinguishing imaging conditions within groups. (**B**) Analyzing the impact of graph-based measurements on LD separation reveals that node strength, degree, and path length showed the largest contribution. LD1 + LD2 together explained 99.6% of variance in the LDA projection. (**C**–**E**) Mahalanobis distance analysis ranked brain structures by their contribution to group separation. (**C**) Brain regions exceeding the reference threshold of one standard deviation (dotted line) were highlighted as prominent contributors to SHAM–PSP separation in RS and LMS. (**D**) Regions separating LMS from RS under SHAM conditions overlapped with those distinguishing SHAM from PSP in RS. Black dots below the x-axis mark regions belonging to the top 10% of Mahalanobis distances, representing the strongest regional contributors to network-state separation across animals. (**E**) PSP animals showed lower average Mahalanobis distances between modalities, indicating reduced modality-dependent separation and higher similarity between RS, LMS, and HMS in the multivariate embedding Black dots below the x-axis mark regions belonging to the top 10% of Mahalanobis distances, representing the strongest regional contributors to network-state separation across animals. RS/LMS-SHAM: *n* = 6; HMS-SHAM: *n* = 7; RS-PSP: *n* = 14; LMS/HMS-PSP: *n* = 17. Node labels are readable in the separately downloadable high-resolution PDF of Fig. 1.

To characterize differences in network organization across groups and imaging conditions, node-specific graph-theoretical parameters were integrated into supervised classification using LDA (Fig. 2). Linear combinations of parameters were calculated as LD dimensions and shown in descending order of their contribution to class separation. We achieved group separation between SHAM and PSP along the first LD dimension, while different imaging conditions were separated along the second LD dimension (Fig. 2A and B). Both LD dimensions combined explained 99.6% of the observed variance and were dominated by the graph-theoretical parameters strength, degree and path length (Fig. 2B). MANOVA analysis of the 2D distribution of both LDs for each group showed significant effects for comparisons between models and imaging conditions (Suppl. Table 2). Cross-validated classification demonstrated moderate discrimination accuracy. Comparing SHAM and PSP animals, 70.9%, 80.9%, and 74.3% of brain structures were correctly classified under resting-state, LMS, and HMS conditions, respectively (Fig. 2A). Comparisons between stimulus conditions and resting-state yielded lower but still above-chance accuracies: 60.9% and 67.4% for LMS and HMS in SHAM animals and 61.7% and 64.8% in PSP animals.

### Mahalanobis distance analysis identifies structures driving group and state separation

To determine which brain structures contributed to separation in LD space, we calculated region-wise Mahalanobis distances (Fig. 2C–E). Larger distances indicate stronger contributions of a given structure to the multivariate separation. As a control comparison, we first examined the model effect (SHAM vs. PSP). Across imaging modalities, the average Mahalanobis distance was lower during HMS compared to resting-state and LMS, indicating that network configurations during high-intensity stimulation were more similar between groups.

Brain structures exceeding the reference threshold of one standard deviation (Fig. 2C, dotted line) were prioritized for interpretation as prominent contributors to SHAM-PSP separation in LMS and RS modalities. Specifically, TeA (temporal association cortex), M2 (secondary motor cortex), and MG (medial geniculate nucleus) contributed to both RS and LMS, while CPiDL (caudate–putamen, dorsolateral), Prh (perirhinal cortex), LG (lateral geniculate nucleus), Cg (cingulate cortex), Re (nucleus reuniens), IC (insular cortex), and Fr (frontal cortex) contributed to RS, and DP (dorsal peduncular cortex), Hip (hippocampus), Amd (amygdala), S1HL (primary somatosensory cortex—hindlimb area), and VPM (ventral posteromedial thalamic nucleus) to LMS discrimination, respectively.

Next, we investigated differences between imaging conditions in both groups separately. In SHAM animals, we identified signatures for LMS (pink-purple, Fig. 2D) and HMS (blue-purple, Fig. 2D) compared to RS, which partly overlapped with those differentiating SHAM and PSP for RS (Fig. 2C). This suggests that LMS and HMS networks in SHAM are shifted toward the PSP resting-state pattern in the multivariate embedding. Moreover, we observed overlap between the signatures distinguishing RS and LMS under SHAM and those distinguishing SHAM and PSP during LMS. In contrast, signatures for imaging conditions under PSP conditions were less pronounced, as reflected by a lower average Mahalanobis distance. This indicates higher similarity between imaging conditions during PSP, compared to the greater dissimilarity observed in SHAM conditions, consistent with reduced imaging-condition-dependent network modulation after incision.

### Stimulus-related Mahalanobis deltas reveal increased dispersion in SHAM networks

To quantify stimulus-related changes in network configuration, we examined absolute Mahalanobis deltas relative to resting-state (RS–LMS and RS–HMS). Across animals and brain regions, the distribution of Mahalanobis deltas differed significantly between groups for both contrasts (KS: dHMS *p* = 0.010; dLMS *p* = 0.004). Notably, the strongest group difference emerged in the dispersion of these delta values. Levene’s test indicated significant variance heterogeneity between groups for the RS–LMS contrast (*p* = 2.6 × 10⁻⁶). Robust dispersion metrics confirmed greater variability in SHAM animals (MAD = 0.876) compared with PSP animals (MAD = 0.797), indicating that stimulus-induced network displacement was more uneven across brain regions in SHAM animals, producing a more “spiky” distribution of regional contributions.

### Regional hotspot analysis confirms focal segregation in SHAM animals

To localize the anatomical origin of this dispersion difference, we examined the frequency with which individual brain regions appeared among the strongest contributors to Mahalanobis deltas. Regions belonging to the top 10% of ΔMah values were identified for each animal and tallied across animals (black dots below x-axis in Fig. 2D and E). This analysis revealed a greater frequency and broader distribution of hotspot regions in SHAM animals, whereas PSP animals showed fewer recurrent peaks and a more diffuse distribution of moderate contributors. The pattern was present for both dHMS and dLMS contrasts and indicates that stimulus-driven network reconfiguration in SHAM animals is characterized by focal regional spikes.

Overall, these analyses demonstrate that stimulus-dependent network modulation is more heterogeneous and regionally specific in SHAM animals, whereas PSP networks show reduced and more spatially distributed multivariate changes.

## Discussion

### Stability of global network topology across fMRI conditions

Our study employed sequential acquisition of rs- and t-fMRI (LMS/HMS) scans under identical conditions, enabling a direct comparison in both experimental groups. Using NBS, a robust method for detecting differences in FC, we did not detect imaging condition-related differences in either group. Similarly, global network properties, including the small-world index and average FC strength, remained unchanged across imaging conditions. These findings indicate that the global organization of networks in the resting-state appears stable and is not readily altered by transient sensory input, consistent with previous reports showing the temporal robustness of these networks (e.g.,^16,23^). Given that mechanical stimulation engages only a limited subset of brain regions, its effect is likely diluted at the whole-brain level, making network-wide differences difficult to detect with NBS. This does not imply that resting and stimulus-induced networks are identical, but rather that differences may be expressed in localized regions or multivariate combinations of node-level features. Consistent with this interpretation, our multivariate Mahalanobis analysis revealed stimulus-related deviations in the latent discriminant space despite the absence of global topology changes. Specifically, RS–LMS and RS–HMS Mahalanobis deltas indicated that imaging-condition effects were expressed as regional shifts in network signatures rather than as global reorganization. Similar findings have been reported in human studies, where rs- and t-fMRI networks largely overlap at the macroscale dimension but diverge in localized sensory and associative areas^5,6,13,27^.

### Loss of fMRI-condition-specific network dynamics in post-surgical pain

To resolve more subtle modality-dependent effects, we applied our graph-theoretical classification framework, which integrates node-level metrics into a multivariate analysis. This approach revealed distinct signatures at the level of individual brain structures^25^. We observed partial overlaps of region rankings discriminating SHAM rs-fMRI from PSP rs-fMRI and SHAM LMS/HMS, suggesting that post-surgery, animals displayed a shift toward enhanced sensorimotor network engagement even at rest. In contrast, LMS/HMS networks in PSP animals were highly similar to their resting networks, indicating a loss of modality-specific processing. This aligns with our previous observation that discrimination between different mechanical stimulus intensities is diminished under post-surgical and inflammatory pain conditions^25^. Human studies have revealed that how much resting and task-related networks differ is influenced by both the type of task and the person’s health, such as in cases of depression or drug use^13,28^. Our findings extend this concept to preclinical fMRI, showing that in animals following surgery the dynamic range of network responses to stimulation is reduced. This interpretation is further supported by the distributional properties of stimulus-related Mahalanobis deltas. In SHAM animals, RS–LMS and RS–HMS contrasts showed greater dispersion across brain regions, indicating that stimulus-driven network shifts were dominated by a limited subset of strongly deviating nodes rather than uniform changes across the network. In contrast, PSP animals displayed significantly lower dispersion of Mahalanobis deltas, particularly for the RS–LMS contrast, suggesting that regional responses became more uniform after surgery. This flattening of regional contributions implies a reduced capacity of the network to selectively recruit specific structures during sensory stimulation. Importantly, dispersion and spatial consistency capture complementary aspects of network organization: dispersion reflects how strongly individual regions deviate, whereas hotspot recurrence indicates whether the same regions consistently drive these deviations across animals.

### Network flexibility and degeneracy as determinants of neural resilience

Our findings are compatible with the idea that unaffected neural networks possess a remarkable degree of resilience and adaptability, enabling them to maintain stable function despite internal fluctuations or external perturbations^29,30^. One proposed mechanism is rooted in the principle of degeneracy, whereby similar network outputs can be achieved through diverse underlying configurations at multiple levels of organization^31–33^. Graph-theoretical work further shows that reconfiguration of community structure, so-called “modular flexibility”, supports adaptive behavior and learning^34^, while network controllability analyses highlight how topology enables transitions between states^35^. Within this framework, modality-dependent reweighting of regional contributions across RS, LMS, and HMS can occur without large changes in global topology. In our data, this corresponds to clearer modality-specific signatures and greater dispersion of regional Mahalanobis contributions in SHAM animals. Post-surgical animals, in contrast, exhibited reduced modality-dependent differentiation and fewer pronounced regional peaks, consistent with a constrained repertoire of accessible network configurations. Consequently, stimulus-driven network modulation in intact animals appears to rely on the flexible recruitment of a limited subset of structures, whereas post-surgical networks show a more homogenized response pattern.

### Pathological consolidation of networks in post-surgical pain

Animals post-surgery showed reduced modality-dependent differentiation, limiting their ability to reconfigure in response to external input. This rigidity can be viewed as a loss of degeneracy, with networks restricted to fewer active configurations and reduced capacity to switch between parallel pathways. Pain-induced changes in synaptic strength, excitability, and stability are well established^36^, and sustained nociceptive drive promotes hyper-excitability and destabilizes network dynamics^37–39^. At the cellular level, long-term synaptic plasticity in pain circuits contributes to the persistence of nociceptive signaling^40,41^, and activity-dependent plasticity has been shown in both spinal and trigeminal relays^42,43^. Such changes can disrupt the excitatory–inhibitory balance and modify circuit gain, effectively compressing the dynamic range of network responses. Consistent with this interpretation, arthritis models show more intense and prolonged brain responses to nociceptive input, producing tighter and less distributed networks^44,45^, while recent post-surgical and inflammatory pain models reveal hypersensitivity-related network signatures and impaired modality discrimination^25^. Pharmacological modulation of spinal GABAergic tone selectively alters stimulus-evoked but not resting cerebral activity^16^ consistent with the differential modulation of stimulus-evoked vs. resting activity. Together with reports of disrupted resting-state dynamics in chronic pain^46,47^, these findings support a pathological consolidation of network states, providing a mechanistic explanation for the reduced variance and dynamic range we observed post-surgery. Our regional Mahalanobis analysis supports this systems-level interpretation. In SHAM animals, stimulus contrasts produced higher dispersion together with recurrent regional peaks among the strongest contributors to network separation, indicating selective and focal recruitment of specific structures. In contrast, PSP animals exhibited fewer consistent hotspot regions and a more uniform distribution of moderate contributors, suggesting that stimulus-related modulation became spatially diffuse rather than driven by distinct network nodes.

Together, these findings indicate a pathological consolidation of network states. Whereas intact networks appear to exploit a broad repertoire of regionally specific configurations, post-surgical networks operate within a more restricted dynamic range, consistent with a loss of degeneracy in the functional network architecture^33^.

### Limitations

First, all scans were acquired under anesthesia, which imposes an experimentally controlled but non-physiological brain state and can alter arousal-related network organization, sensory responsiveness, and neurovascular coupling^23,48^. Accordingly, our conclusions should be interpreted as comparisons of resting-state and stimulation-evoked network organization within this standardized anesthetized regimen, rather than as direct statements about awake network dynamics. Second, stimulation runs were acquired sequentially (LMS followed by HMS). This design introduces practical confounds of potential order effects (time-in-scanner, habituation/sensitization). Importantly, these factors are expected to affect both groups and may reduce apparent RS–HMS differentiation irrespective of injury status; thus, HMS-related “similarity” should be interpreted cautiously as it may reflect ceiling effects in addition to pain-state physiology. Third, the within-subject dataset was not fully balanced across modalities for all animals, which reduces power for paired inference and contributes to wider uncertainty, most notably for RS–LP comparisons in SHAM, so effects that are directionally consistent may remain difficult to resolve statistically in the smallest paired strata. Finally, our multivariate analyses rely on predefined anatomical parcellations and linear discriminant representations of graph-theoretical features. While this approach enhances sensitivity to distributed network signatures, it assumes stable regional boundaries and linear separability in latent space, which may not capture all aspects of nonlinear network dynamics.

## Conclusion

Our results show that global network topology remained broadly similar across resting-state and stimulation fMRI conditions, whereas post-surgical pain alters how networks reconfigure in response to sensory input. Multivariate analyses revealed that SHAM animals exhibit stimulus-dependent regional specialization characterized by heterogeneous Mahalanobis contributions and focal regional hotspots. In contrast, PSP animals showed reduced dispersion and diminished regional differentiation, indicating a more uniform pattern of network modulation.

These findings suggest that pain-related network dysfunction is primarily expressed as reduced flexibility of regional network responses rather than large-scale changes in global topology. Integrating resting-state and stimulus-based fMRI therefore provides complementary insight into disease-related alterations in brain network dynamics that remain undetected by resting-state analysis alone.

## Electronic Supplementary Material

Below is the link to the electronic supplementary material.

Supplementary Material 1

## Data Availability

Data are publicly available under https://openneuro.org/datasets/ds005839/versions/1.0.1.
