# Supplementary material for: Integration of resting-state and stimulus-fMRI uncovers reduced network flexibility in post-surgical pain
Source: Sci Rep. 2026 May 19;16:15570. doi: 10.1038/s41598-026-51946-5 (PMC13187255; doi:10.1038/s41598-026-51946-5)
Supplement: Supplementary file 1 — Supplementary Material 1 [file 41598_2026_51946_MOESM1_ESM.pdf]

Supplemental Material

Table 1: List of Structures of Rat Brain Atlas.

Table 2: MANOVA analysis of 2D distribution of LD1 and LD2 for each brain structure across model and imaging condition.

Suppl. Table 1. ½: List of Structures of Rat Brain Atlas.

ID	Structure Name	Hemisphere	Functional Group
Cg li	cingulate cortex, links	li	association cortex
Cg re	cingulate cortex, rechts	re	association cortex
DP li	dorsal peduncular cortex, links	li	association cortex
DP re	dorsal peduncular cortex, rechts	re	association cortex
Fr3 li	frotal cortex area 3, links	li	association cortex
Fr3 re	frotal cortex area 3, rechts	re	association cortex
FrA li	frontal association cortex, links	li	association cortex
FrA re	frontal association cortex, rechts	re	association cortex
IL li	infralimbic cortex, links	li	association cortex
IL re	infralimbic cortex, rechts	re	association cortex
Ins li	insular cortex, links	li	association cortex
Ins re	insular cortex, rechts	re	association cortex
Orb li	orbital cortex, links	li	association cortex
Orb re	orbital cortex, rechts	re	association cortex
PrL li	prelimbic cortex, links	li	association cortex
PrL re	prelimbic cortex, rechts	re	association cortex
PtA li	parietal association cortex, links	li	association cortex
PtA re	parietal association cortex, rechts	re	association cortex
RS li	retrosplenial cortex, links	li	association cortex
RS re	retrosplenial cortex, rechts	re	association cortex
TeA li	temporal association cortex, links	li	association cortex
TeA re	temporal association cortex, rechts	re	association cortex
Acb li	nucleus accumbens, links	li	basalganglia
Acb re	nucleus accumbens, rechts	re	basalganglia
Cl li	claustrum, links	li	basalganglia
Cl re	claustrum, rechts	re	basalganglia
CPc li	Caudales Striatum, links	li	basalganglia
CPc re	Caudales Striatum, rechts	re	basalganglia
CPiDL li	Dorsolaterales Intermediäres Striatum, links	li	basalganglia
CPiDL re	Dorsolaterales Intermediäres Striatum, rechts	re	basalganglia
CPiDM li	Dorsomediales Intermediäres Striatum, links	li	basalganglia
CPiDM re	Dorsomediales Intermediäres Striatum, rechts	re	basalganglia
CPiVL li	Ventrolaterales Intermediäres Striatum, links	li	basalganglia
CPiVL re	Ventrolaterales Intermediäres Striatum, rechts	re	basalganglia
CPiVM li	Ventromediales Intermediäres Striatum, links	li	basalganglia
CPiVM re	Ventromediales Intermediäres Striatum, rechts	re	basalganglia
CPr li	Rostrales Striatum, links	li	basalganglia
CPr re	Rostrales Striatum, rechts	re	basalganglia
GP li	globus pallidus, links	li	basalganglia
GP re	globus pallidus, rechts	re	basalganglia
VP li	ventral pallidum, links	li	basalganglia
VP re	ventral pallidum, rechts	re	basalganglia
HT li	hypothalamus, links	li	limbic output
HT re	hypothalamus, rechts	re	limbic output
PAG	periaqueductal gray	m	limbic output
ZI li	zona incerta, links	li	limbic output
ZI re	zona incerta, rechts	re	limbic output
Amd li	amygdala, links	li	limbic system
Amd re	amygdala, rechts	re	limbic system
BST li	bed nucleus of stria terminalis, links	li	limbic system
BST re	bed nucleus of stria terminalis, rechts	re	limbic system
CoM	corpora mammillaria	m	limbic system
DB li	nuclei of diagonal band, links	li	limbic system
DB re	nuclei of diagonal band, rechts	re	limbic system
Hb li	habenuli, links	li	limbic system
Hb re	habenuli, rechts	re	limbic system
Hip li	hippocampus, links	li	limbic system
Hip re	hippocampus, rechts	re	limbic system
Sep li	septal area, links	li	limbic system
Sep re	septal area, rechts	re	limbic system
SLEA li	sublenticular extended amygdala, links	li	limbic system
SLEA re	sublenticular extended amygdala, rechts	re	limbic system
Ent li	entorhinal cortex, links	li	link to the limbic system
Ent re	entorhinal cortex, rechts	re	link to the limbic system
Pir li	piriform cortex, links	li	link to the limbic system
Pir re	piriform cortex, rechts	re	link to the limbic system
Prh_Ect li	perirhinal/ectorhinal cortex, links	li	link to the limbic system
Prh_Ect re	perirhinal/ectorhinal cortex, rechts	re	link to the limbic system

Suppl. Table 1. 2/2

ID	Structure Name	Hemisphere	Functional Group
Cb li	cerebellum, links	li	motor output
Cb re	cerebellum, rechts	re	motor output
M2 re	secondary motor cortex, rechts	re	motor output
M1 li	primary motor cortex, links	li	motoric output
M1 re	primary motor cortex, rechts	re	motoric output
M2 li	secondary motor cortex, links	li	motoric output
S1HL li	primary somatosensory cortex hind limb, links	li	sensory cortex
S1HL re	primary somatosensory cortex hind limb, rechts	re	sensory cortex
S1r li	primary somatosensory cortex rest, links	li	sensory cortex
S1r re	primary somatosensory cortex rest, rechts	re	sensory cortex
S2 li	secondary somatosensory cortex, links	li	sensory cortex
S2 re	secondary somatosensory cortex, rechts	re	sensory cortex
CnF li	cuneiform nucleus, links	li	sensory input
CnF re	cuneiform nucleus, rechts	re	sensory input
Gi li	gigantocellular reticular nucleus, links	li	sensory input
Gi re	gigantocellular reticular nucleus, rechts	re	sensory input
IC li	inferior colliculus, links	li	sensory input
IC re	inferior colliculus, rechts	re	sensory input
IP	interpeduncular nucleus	m	sensory input
LPB li	lateral parabrachial nucleus, links	li	sensory input
LPB re	lateral parabrachial nucleus, rechts	re	sensory input
LPGi li	lateral paragigantocellular nucleus, links	li	sensory input
LPGi re	lateral paragigantocellular nucleus, rechts	re	sensory input
LRt li	lateral reticular nucleus, links	li	sensory input
LRt re	lateral reticular nucleus, rechts	re	sensory input
MdD li	dorsal medullary reticular nucleus, links	li	sensory input
MdD re	dorsal medullary reticular nucleus, rechts	re	sensory input
MdV li	ventral medullary reticular nucleus, links	li	sensory input
MdV re	ventral medullary reticular nucleus, rechts	re	sensory input
PCRt li	parvicellular reticular nucleus, links	li	sensory input
PCRt re	parvicellular reticular nucleus, rechts	re	sensory input
Pn li	pontine reticular nucleus, links	li	sensory input
Pn re	pontine reticular nucleus, rechts	re	sensory input
PTA li	pretectal area, links	li	sensory input
PTA re	pretectal area, rechts	re	sensory input
R	raphe nucleus	m	sensory input
Re li	red nucleus, links	li	sensory input
Re re	red nucleus, rechts	re	sensory input
SC li	superior colliculus, links	li	sensory input
SC re	superior colliculus, rechts	re	sensory input
Teg li	tegmental nuclei, links	li	sensory input
Teg re	tegmental nuclei, rechts	re	sensory input
VTA li	ventral tegmental area, links	li	sensory input
VTA re	ventral tegmental area, rechts	re	sensory input
Ant li	anterior thalamic group, links	li	thalamus
Ant re	anterior thalamic group, rechts	re	thalamus
LG li	lateral geniculate nucleus, links	li	thalamus
LG re	lateral geniculate nucleus, rechts	re	thalamus
LP li	lateral posterior thalamic nucleus, links	li	thalamus
LP re	lateral posterior thalamic nucleus, rechts	re	thalamus
MG li	medial geniculate nucleus, links	li	thalamus
MG re	medial geniculate nucleus, rechts	re	thalamus
MT li	medial thalamus, links	li	thalamus
MT re	medial thalamus, rechts	re	thalamus
Po li	posterior thalamic nuclear group, links	li	thalamus
Po re	posterior thalamic nuclear group, rechts	re	thalamus
PV	paraventricular thalamic nucleus	m	thalamus
Rt li	reticular thalamic nucleus, links	li	thalamus
Rt re	reticular thalamic nucleus, rechts	re	thalamus
Sub li	submedius thalamic nucleus, links	li	thalamus
Sub re	submedius thalamic nucleus, rechts	re	thalamus
VA_VL li	thalamic nucleus ventralis anterolateralis, links	li	thalamus
VA_VL re	thalamic nucleus ventralis anterolateralis, rechts	re	thalamus
VM li	ventromedial thalamic nucleus, links	li	thalamus
VM re	ventromedial thalamic nucleus, rechts	re	thalamus
VPL_VPM li	ventral postolateral/posteromedial thalamic nucleus, links	li	thalamus
VPL_VPM re	ventral postolateral/posteromedial thalamic nucleus, rechts	re	thalamus

Suppl. Table 2. MANOVA analysis of 2D distribution of LD1 and LD2 for each brain structure across model and imaging condition.

**Model**	**fMRI condition**	**sig.**	**Wilks-Lambda**
SHAM vs. PSP	ALL	< 0.001	0.633
SHAM vs. PSP	RS	< 0.001	0.735
SHAM vs. PSP	LMS	< 0.001	0.622
SHAM vs. PSP	HMS	< 0.001	0.727
SHAM	ALL	< 0.001	0.877
SHAM	RS vs. LMS	0.003	0.951
SHAM	RS vs. HMS	< 0.001	0.846
SHAM	LMS vs. HMS	< 0.001	0.925
PSP	ALL	< 0.001	0.866
PSP	RS vs. LMS	< 0.001	0.920
PSP	RS vs. HMS	< 0.001	0.865
PSP	LMS vs. HMS	< 0.001	0.914
